# Methyl 5-chloro-2-[*N*-(3-eth­oxy­carbonyl­prop­yl)-4-methyl­benzene­sulfonamido]­benzoate

**DOI:** 10.1107/S1600536810023792

**Published:** 2010-06-26

**Authors:** Bin Wang, Ru Jia, Ya-Bin Shi, Fei-Fei He, Hai-Bo Wang

**Affiliations:** a College of Food Science and Light Industry, Nanjing University of Technology, Xinmofan Road No.5 Nanjing, Nanjing 210009, People’s Republic of China; b College of Science, Nanjing University of Technology, Xinmofan Road No.5 Nanjing, Nanjing 210009, People’s Republic of China

## Abstract

In the title compound, C_21_H_24_ClNO_6_S, the benzene rings are oriented at a dihedral angles of 41.6 (2)°. In the crystal structure, weak inter­molecular C—H⋯O inter­actions link the mol­ecules.

## Related literature

For the preparation of the title compound, see: Kondo *et al.* (1999 ▶). For bond-length data, see: Allen *et al.* (1987 ▶).
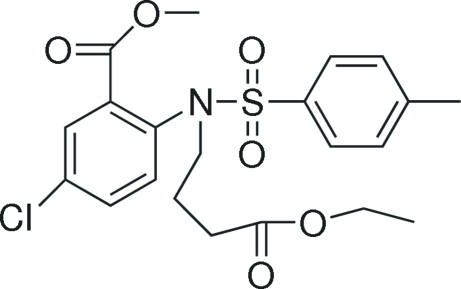


## Experimental

### 

#### Crystal data

C_21_H_24_ClNO_6_S
*M* *_r_* = 453.92
Orthorhombic, 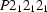

*a* = 9.1480 (18) Å
*b* = 10.742 (2) Å
*c* = 23.258 (5) Å
*V* = 2285.5 (8) Å^3^
*Z* = 4
Mo *K*α radiation
μ = 0.29 mm^−1^
*T* = 296 K
0.30 × 0.10 × 0.05 mm

#### Data collection

Enraf–Nonius CAD-4 diffractometer
Absorption correction: ψ scan (North *et al.*, 1968 ▶) *T*_min_ = 0.917, *T*_max_ = 0.985
4623 measured reflections
4130 independent reflections
2134 reflections with *I* > 2σ(*I*)
*R*_int_ = 0.072
3 standard reflections every 200 reflections  intensity decay: 1%

#### Refinement

*R*[*F* ^2^ > 2σ(*F* ^2^)] = 0.063
*wR*(*F* ^2^) = 0.114
*S* = 0.92
4130 reflections
271 parameters
H-atom parameters constrained
Δρ_max_ = 0.19 e Å^−3^
Δρ_min_ = −0.21 e Å^−3^
Absolute structure: Flack (1983 ▶), 1748 Friedel pairs
Flack parameter: 0.03 (12)

### 

Data collection: *CAD-4 EXPRESS* (Enraf–Nonius, 1989 ▶); cell refinement: *CAD-4 EXPRESS*; data reduction: *XCAD4* (Harms & Wocadlo, 1995 ▶); program(s) used to solve structure: *SHELXS97* (Sheldrick, 2008 ▶); program(s) used to refine structure: *SHELXL97* (Sheldrick, 2008 ▶); molecular graphics: *SHELXTL* (Sheldrick, 2008 ▶); software used to prepare material for publication: *PLATON* (Spek, 2009 ▶).

## Supplementary Material

Crystal structure: contains datablocks global, I. DOI: 10.1107/S1600536810023792/bq2221sup1.cif

Structure factors: contains datablocks I. DOI: 10.1107/S1600536810023792/bq2221Isup2.hkl

Additional supplementary materials:  crystallographic information; 3D view; checkCIF report

## Figures and Tables

**Table 1:** Hydrogen-bond geometry (Å, °)

*D*—H⋯*A*	*D*—H	H⋯*A*	*D*⋯*A*	*D*—H⋯*A*
C11—H11*A*⋯O3^i^	0.93	2.55	3.275 (6)	135
C14—H14*A*⋯O2^ii^	0.96	2.60	3.338 (7)	134
C17—H17*A*⋯O2^iii^	0.93	2.59	3.414 (8)	148

Symmetry codes: (i) 
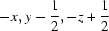
; (ii) 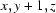; (iii) 
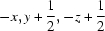
.

**Fig. 1.**
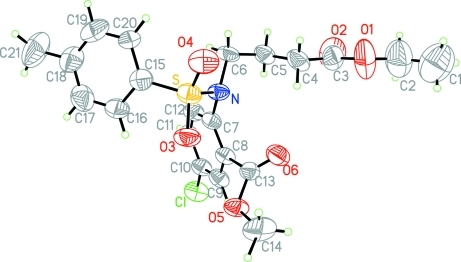
The molecular structure of the title molecule, with the atom-numbering scheme. Displacement ellipsoids are drawn at the 50% probability level.

## supplementary crystallographic information

### Comment

Quinolines are a major class of alkaloids and play an important role in the fields of natural products and medicinal chemistry. The title compound, (I), is a useful intermediate. (Kondo *et al.*, 1999). In the molecule of the title compound (Fig. 1), the bond lengths (Allen *et al.*,  1987) and angles are within normal ranges. The intramolecular C-H···O hydrogen bond (Table 1) results in the formation of a five-membered ring C (C6/H6A/O4/S/N). Rings A(C7-C12) and B(C15-C20) are planar with maximum deviations of 0.011 (4) Å for C9 and -0.021 (5) Å for C16, respectively. The dihedral angle between these rings is 41.6 (2) °. In the crystal structure, intermolecular weak C-H..O interactions link the molecules to form a stable structure.

### Experimental

The title compound, (I) was prepared by the literature method (Kondo *et al.*, 1999). Crystals suitable for X-ray analysis were obtained by slow evaporation of an methanol solution.

### Refinement

H atoms were positioned geometrically, with C-H =0.93, 0.98 and 0.96 Å for aromatic, methine and methyl H, respectively, and constrained to ride on their parent atoms, with U_iso_(H) = xU_eq_(C,N), where x = 1.5 for methyl H and x = 1.2 for all other H atoms.

### Crystal data

**Table:**

C_21_H_24_ClNO_6_S	*D*_x_ = 1.319 Mg m^−^^3^
*M**_r_* = 453.92	Melting point: 353 K
Orthorhombic, *P*2_1_2_1_2_1_	Mo *K*α radiation, λ = 0.71073 Å
Hall symbol: P 2ac 2ab	Cell parameters from 25 reflections
*a* = 9.1480 (18) Å	θ = 8–12°
*b* = 10.742 (2) Å	µ = 0.29 mm^−^^1^
*c* = 23.258 (5) Å	*T* = 296 K
*V* = 2285.5 (8) Å^3^	Needle, colorless
*Z* = 4	0.30 × 0.10 × 0.05 mm
*F*(000) = 952	

### Data collection

**Table:**

Enraf–Nonius CAD-4 diffractometer	2134 reflections with *I* > 2σ(*I*)
Radiation source: fine-focus sealed tube	*R*_int_ = 0.072
graphite	θ_max_ = 25.3°, θ_min_ = 1.8°
ω/2θ scans	*h* = −10→0
Absorption correction: ψ scan (North *et al.*, 1968)	*k* = −12→0
*T*_min_ = 0.917, *T*_max_ = 0.985	*l* = −27→27
4623 measured reflections	3 standard reflections every 200 reflections
4130 independent reflections	intensity decay: 1%

### Refinement

**Table:**

Refinement on *F*^2^	Secondary atom site location: difference Fourier map
Least-squares matrix: full	Hydrogen site location: inferred from neighbouring sites
*R*[*F*^2^ > 2σ(*F*^2^)] = 0.063	H-atom parameters constrained
*wR*(*F*^2^) = 0.114	*w* = 1/[σ^2^(*F*_o_^2^) + (0.025*P*)^2^] where *P* = (*F*_o_^2^ + 2*F*_c_^2^)/3
*S* = 0.92	(Δ/σ)_max_ < 0.001
4130 reflections	Δρ_max_ = 0.19 e Å^−^^3^
271 parameters	Δρ_min_ = −0.21 e Å^−^^3^
0 restraints	Absolute structure: Flack (1983), 1748 Friedel pairs
Primary atom site location: structure-invariant direct methods	Flack parameter: 0.03 (12)

### Special details

**Table:**

Geometry. All esds (except the esd in the dihedral angle between two l.s. planes) are estimated using the full covariance matrix. The cell esds are taken into account individually in the estimation of esds in distances, angles and torsion angles; correlations between esds in cell parameters are only used when they are defined by crystal symmetry. An approximate (isotropic) treatment of cell esds is used for estimating esds involving l.s. planes.
Refinement. Refinement of F^2^ against ALL reflections. The weighted R-factor wR and goodness of fit S are based on F^2^, conventional R-factors R are based on F, with F set to zero for negative F^2^. The threshold expression of F^2^ > 2sigma(F^2^) is used only for calculating R-factors(gt) etc. and is not relevant to the choice of reflections for refinement. R-factors based on F^2^ are statistically about twice as large as those based on F, and R- factors based on ALL data will be even larger.

### Fractional atomic coordinates and isotropic or equivalent isotropic displacement parameters (Å^2^)

**Table:**

	*x*	*y*	*z*	*U*_iso_*/*U*_eq_	
S	0.32206 (15)	0.97963 (12)	0.21916 (6)	0.0523 (4)	
Cl	−0.34814 (14)	1.01035 (14)	0.35104 (7)	0.0753 (5)	
O1	0.5372 (6)	0.6826 (5)	0.4389 (3)	0.130 (2)	
O2	0.3409 (6)	0.5726 (4)	0.4257 (2)	0.1119 (19)	
O3	0.2927 (4)	1.1049 (3)	0.23835 (15)	0.0605 (11)	
O4	0.4681 (4)	0.9398 (3)	0.21051 (16)	0.0668 (12)	
O5	0.1790 (4)	1.2067 (3)	0.35132 (15)	0.0537 (9)	
O6	0.3165 (4)	1.0402 (3)	0.37290 (16)	0.0651 (11)	
N	0.2482 (4)	0.8844 (3)	0.26778 (18)	0.0459 (12)	
C1	0.6798 (12)	0.6647 (8)	0.5254 (4)	0.165 (4)	
H1A	0.6820	0.6204	0.5613	0.247*	
H1B	0.6714	0.7524	0.5328	0.247*	
H1C	0.7684	0.6487	0.5045	0.247*	
C2	0.5609 (11)	0.6252 (9)	0.4933 (4)	0.145 (4)	
H2A	0.4737	0.6372	0.5163	0.174*	
H2B	0.5713	0.5363	0.4869	0.174*	
C3	0.4202 (8)	0.6527 (6)	0.4104 (3)	0.077 (2)	
C4	0.4113 (6)	0.7278 (5)	0.3548 (3)	0.0671 (17)	
H4B	0.3982	0.8152	0.3641	0.080*	
H4C	0.5023	0.7193	0.3338	0.080*	
C5	0.2883 (6)	0.6854 (5)	0.3181 (2)	0.0570 (16)	
H5A	0.1971	0.6994	0.3384	0.068*	
H5B	0.2978	0.5966	0.3116	0.068*	
C6	0.2816 (6)	0.7504 (4)	0.2610 (2)	0.0553 (16)	
H6A	0.3746	0.7411	0.2415	0.066*	
H6B	0.2069	0.7118	0.2374	0.066*	
C7	0.1045 (6)	0.9196 (4)	0.2868 (2)	0.0452 (13)	
C8	0.0844 (5)	1.0121 (4)	0.3272 (2)	0.0412 (12)	
C9	−0.0552 (5)	1.0403 (5)	0.3460 (2)	0.0543 (15)	
H9A	−0.0678	1.1043	0.3725	0.065*	
C10	−0.1762 (6)	0.9764 (4)	0.3267 (2)	0.0549 (14)	
C11	−0.1565 (6)	0.8815 (5)	0.2868 (3)	0.0610 (16)	
H11A	−0.2360	0.8361	0.2734	0.073*	
C12	−0.0171 (6)	0.8557 (5)	0.2676 (3)	0.0562 (16)	
H12A	−0.0045	0.7927	0.2405	0.067*	
C13	0.2085 (6)	1.0834 (4)	0.3535 (2)	0.0476 (13)	
C14	0.2962 (6)	1.2856 (5)	0.3736 (3)	0.080 (2)	
H14A	0.2676	1.3714	0.3709	0.120*	
H14B	0.3834	1.2724	0.3514	0.120*	
H14C	0.3147	1.2648	0.4131	0.120*	
C15	0.2247 (6)	0.9560 (5)	0.1556 (2)	0.0558 (15)	
C16	0.0987 (6)	1.0246 (6)	0.1445 (3)	0.0737 (18)	
H16A	0.0677	1.0842	0.1708	0.088*	
C17	0.0199 (7)	1.0052 (7)	0.0952 (3)	0.082 (2)	
H17A	−0.0607	1.0549	0.0870	0.098*	
C18	0.0601 (7)	0.9126 (7)	0.0581 (3)	0.074 (2)	
C19	0.1794 (8)	0.8414 (6)	0.0698 (3)	0.078 (2)	
H19A	0.2045	0.7768	0.0451	0.094*	
C20	0.2628 (7)	0.8637 (6)	0.1175 (2)	0.0659 (18)	
H20A	0.3460	0.8160	0.1242	0.079*	
C21	−0.0298 (8)	0.8910 (8)	0.0048 (3)	0.132 (3)	
H21A	−0.1074	0.9511	0.0032	0.198*	
H21B	−0.0705	0.8087	0.0059	0.198*	
H21C	0.0311	0.8995	−0.0285	0.198*	

### Atomic displacement parameters (Å^2^)

**Table:**

	*U*^11^	*U*^22^	*U*^33^	*U*^12^	*U*^13^	*U*^23^
S	0.0493 (8)	0.0417 (7)	0.0659 (10)	−0.0006 (8)	0.0099 (8)	0.0019 (8)
Cl	0.0481 (8)	0.0653 (10)	0.1124 (13)	−0.0118 (8)	0.0152 (9)	−0.0006 (10)
O1	0.139 (5)	0.116 (4)	0.134 (5)	−0.032 (4)	−0.076 (4)	0.041 (4)
O2	0.134 (5)	0.089 (4)	0.113 (4)	−0.045 (4)	−0.014 (4)	0.026 (3)
O3	0.084 (3)	0.0296 (18)	0.068 (3)	0.002 (2)	0.012 (2)	−0.0045 (17)
O4	0.054 (2)	0.063 (3)	0.083 (3)	−0.006 (2)	0.017 (2)	−0.006 (2)
O5	0.046 (2)	0.0352 (18)	0.080 (3)	−0.0061 (18)	−0.001 (2)	−0.0094 (19)
O6	0.056 (2)	0.051 (2)	0.088 (3)	0.009 (2)	−0.016 (2)	−0.007 (2)
N	0.055 (3)	0.031 (2)	0.051 (3)	0.000 (2)	0.014 (2)	0.001 (2)
C1	0.192 (12)	0.155 (9)	0.148 (9)	−0.042 (9)	−0.011 (9)	−0.002 (7)
C2	0.163 (10)	0.136 (8)	0.138 (9)	−0.013 (8)	−0.045 (8)	0.033 (7)
C3	0.087 (6)	0.056 (4)	0.087 (5)	−0.010 (4)	−0.028 (5)	−0.004 (4)
C4	0.070 (4)	0.041 (3)	0.090 (5)	0.008 (3)	−0.011 (4)	0.007 (4)
C5	0.057 (4)	0.039 (3)	0.075 (4)	0.002 (3)	−0.005 (3)	0.004 (3)
C6	0.068 (4)	0.029 (3)	0.070 (4)	0.013 (3)	0.007 (3)	−0.001 (3)
C7	0.046 (3)	0.033 (3)	0.056 (4)	0.000 (3)	0.005 (3)	0.007 (3)
C8	0.034 (3)	0.030 (3)	0.060 (3)	−0.008 (3)	0.003 (2)	0.004 (3)
C9	0.046 (3)	0.040 (3)	0.077 (4)	−0.004 (3)	−0.006 (3)	0.006 (3)
C10	0.050 (3)	0.039 (3)	0.076 (4)	−0.007 (3)	0.002 (3)	0.003 (3)
C11	0.052 (4)	0.051 (3)	0.080 (4)	0.000 (3)	−0.011 (4)	0.003 (3)
C12	0.057 (4)	0.039 (3)	0.072 (5)	−0.005 (3)	−0.004 (3)	−0.002 (3)
C13	0.053 (4)	0.040 (3)	0.050 (3)	0.001 (3)	0.000 (3)	−0.002 (3)
C14	0.068 (4)	0.050 (3)	0.122 (6)	−0.032 (4)	0.002 (4)	−0.019 (4)
C15	0.059 (4)	0.050 (3)	0.058 (4)	−0.004 (3)	0.010 (3)	0.000 (3)
C16	0.067 (4)	0.082 (5)	0.073 (5)	0.036 (4)	0.004 (4)	−0.009 (4)
C17	0.063 (4)	0.102 (6)	0.081 (5)	0.018 (5)	0.001 (4)	0.017 (5)
C18	0.051 (4)	0.107 (6)	0.065 (5)	−0.003 (4)	0.010 (4)	−0.001 (5)
C19	0.084 (5)	0.082 (5)	0.069 (5)	−0.010 (5)	0.030 (4)	−0.017 (4)
C20	0.081 (5)	0.068 (4)	0.049 (4)	0.029 (4)	0.013 (4)	−0.005 (3)
C21	0.113 (7)	0.215 (10)	0.068 (5)	−0.005 (7)	−0.021 (5)	−0.025 (6)

### Geometric parameters (Å, °)

**Table:**

S—O4	1.417 (4)	C7—C8	1.380 (6)
S—O3	1.443 (3)	C7—C12	1.382 (7)
S—N	1.668 (4)	C8—C9	1.383 (6)
S—C15	1.745 (6)	C8—C13	1.500 (7)
Cl—C10	1.711 (5)	C9—C10	1.377 (6)
O1—C3	1.299 (7)	C9—H9A	0.9300
O1—C2	1.424 (9)	C10—C11	1.390 (7)
O2—C3	1.181 (7)	C11—C12	1.380 (7)
O5—C13	1.353 (5)	C11—H11A	0.9300
O5—C14	1.461 (6)	C12—H12A	0.9300
O6—C13	1.181 (6)	C14—H14A	0.9600
N—C7	1.437 (6)	C14—H14B	0.9600
N—C6	1.480 (5)	C14—H14C	0.9600
C1—C2	1.387 (10)	C15—C20	1.375 (7)
C1—H1A	0.9600	C15—C16	1.392 (7)
C1—H1B	0.9600	C16—C17	1.371 (8)
C1—H1C	0.9600	C16—H16A	0.9300
C2—H2A	0.9700	C17—C18	1.367 (8)
C2—H2B	0.9700	C17—H17A	0.9300
C3—C4	1.525 (8)	C18—C19	1.360 (8)
C4—C5	1.484 (7)	C18—C21	1.504 (8)
C4—H4B	0.9700	C19—C20	1.368 (8)
C4—H4C	0.9700	C19—H19A	0.9300
C5—C6	1.501 (7)	C20—H20A	0.9300
C5—H5A	0.9700	C21—H21A	0.9600
C5—H5B	0.9700	C21—H21B	0.9600
C6—H6A	0.9700	C21—H21C	0.9600
C6—H6B	0.9700		
			
O4—S—O3	120.1 (2)	C7—C8—C13	123.0 (4)
O4—S—N	107.0 (2)	C9—C8—C13	117.3 (5)
O3—S—N	106.7 (2)	C10—C9—C8	122.0 (5)
O4—S—C15	108.5 (3)	C10—C9—H9A	119.0
O3—S—C15	107.6 (2)	C8—C9—H9A	119.0
N—S—C15	106.2 (2)	C9—C10—C11	118.6 (5)
C3—O1—C2	118.1 (7)	C9—C10—Cl	121.6 (4)
C13—O5—C14	114.1 (4)	C11—C10—Cl	119.7 (4)
C7—N—C6	118.5 (4)	C12—C11—C10	118.9 (5)
C7—N—S	114.7 (3)	C12—C11—H11A	120.5
C6—N—S	116.1 (3)	C10—C11—H11A	120.5
C2—C1—H1A	109.5	C11—C12—C7	122.6 (5)
C2—C1—H1B	109.5	C11—C12—H12A	118.7
H1A—C1—H1B	109.5	C7—C12—H12A	118.7
C2—C1—H1C	109.5	O6—C13—O5	124.5 (5)
H1A—C1—H1C	109.5	O6—C13—C8	126.0 (5)
H1B—C1—H1C	109.5	O5—C13—C8	109.5 (5)
C1—C2—O1	117.8 (9)	O5—C14—H14A	109.5
C1—C2—H2A	107.9	O5—C14—H14B	109.5
O1—C2—H2A	107.9	H14A—C14—H14B	109.5
C1—C2—H2B	107.9	O5—C14—H14C	109.5
O1—C2—H2B	107.9	H14A—C14—H14C	109.5
H2A—C2—H2B	107.2	H14B—C14—H14C	109.5
O2—C3—O1	122.2 (7)	C20—C15—C16	118.2 (6)
O2—C3—C4	127.4 (7)	C20—C15—S	121.4 (5)
O1—C3—C4	110.2 (6)	C16—C15—S	120.1 (5)
C5—C4—C3	111.5 (5)	C17—C16—C15	120.6 (6)
C5—C4—H4B	109.3	C17—C16—H16A	119.7
C3—C4—H4B	109.3	C15—C16—H16A	119.7
C5—C4—H4C	109.3	C18—C17—C16	119.8 (6)
C3—C4—H4C	109.3	C18—C17—H17A	120.1
H4B—C4—H4C	108.0	C16—C17—H17A	120.1
C4—C5—C6	113.4 (5)	C19—C18—C17	119.9 (7)
C4—C5—H5A	108.9	C19—C18—C21	121.1 (7)
C6—C5—H5A	108.9	C17—C18—C21	119.0 (7)
C4—C5—H5B	108.9	C18—C19—C20	120.8 (6)
C6—C5—H5B	108.9	C18—C19—H19A	119.6
H5A—C5—H5B	107.7	C20—C19—H19A	119.6
N—C6—C5	111.5 (4)	C19—C20—C15	120.5 (6)
N—C6—H6A	109.3	C19—C20—H20A	119.8
C5—C6—H6A	109.3	C15—C20—H20A	119.8
N—C6—H6B	109.3	C18—C21—H21A	109.5
C5—C6—H6B	109.3	C18—C21—H21B	109.5
H6A—C6—H6B	108.0	H21A—C21—H21B	109.5
C8—C7—C12	118.1 (5)	C18—C21—H21C	109.5
C8—C7—N	121.4 (4)	H21A—C21—H21C	109.5
C12—C7—N	120.4 (5)	H21B—C21—H21C	109.5
C7—C8—C9	119.7 (5)		
			
O4—S—N—C7	173.3 (4)	C9—C10—C11—C12	−0.8 (8)
O3—S—N—C7	43.6 (4)	Cl—C10—C11—C12	179.9 (4)
C15—S—N—C7	−71.0 (4)	C10—C11—C12—C7	0.8 (9)
O4—S—N—C6	−42.5 (4)	C8—C7—C12—C11	0.5 (8)
O3—S—N—C6	−172.2 (4)	N—C7—C12—C11	176.5 (5)
C15—S—N—C6	73.2 (4)	C14—O5—C13—O6	2.0 (8)
C3—O1—C2—C1	−175.0 (9)	C14—O5—C13—C8	−177.4 (4)
C2—O1—C3—O2	−6.6 (12)	C7—C8—C13—O6	−47.9 (8)
C2—O1—C3—C4	177.6 (7)	C9—C8—C13—O6	130.6 (6)
O2—C3—C4—C5	−2.1 (10)	C7—C8—C13—O5	131.6 (5)
O1—C3—C4—C5	173.4 (6)	C9—C8—C13—O5	−49.9 (6)
C3—C4—C5—C6	−175.8 (5)	O4—S—C15—C20	28.1 (5)
C7—N—C6—C5	−69.4 (6)	O3—S—C15—C20	159.4 (4)
S—N—C6—C5	147.8 (4)	N—S—C15—C20	−86.6 (5)
C4—C5—C6—N	−65.9 (6)	O4—S—C15—C16	−157.0 (4)
C6—N—C7—C8	137.4 (5)	O3—S—C15—C16	−25.7 (5)
S—N—C7—C8	−79.3 (5)	N—S—C15—C16	88.2 (5)
C6—N—C7—C12	−38.6 (7)	C20—C15—C16—C17	−3.8 (9)
S—N—C7—C12	104.8 (5)	S—C15—C16—C17	−178.8 (5)
C12—C7—C8—C9	−1.6 (7)	C15—C16—C17—C18	4.0 (10)
N—C7—C8—C9	−177.7 (5)	C16—C17—C18—C19	−1.0 (10)
C12—C7—C8—C13	176.8 (5)	C16—C17—C18—C21	178.6 (6)
N—C7—C8—C13	0.8 (7)	C17—C18—C19—C20	−2.1 (10)
C7—C8—C9—C10	1.7 (8)	C21—C18—C19—C20	178.3 (6)
C13—C8—C9—C10	−176.9 (5)	C18—C19—C20—C15	2.2 (9)
C8—C9—C10—C11	−0.4 (8)	C16—C15—C20—C19	0.7 (8)
C8—C9—C10—Cl	178.9 (4)	S—C15—C20—C19	175.7 (5)

### Hydrogen-bond geometry (Å, °)

**Table:**

*D*—H···*A*	*D*—H	H···*A*	*D*···*A*	*D*—H···*A*
C6—H6A···O4	0.97	2.41	2.903 (6)	111
C11—H11A···O3^i^	0.93	2.55	3.275 (6)	135
C14—H14A···O2^ii^	0.96	2.60	3.338 (7)	134
C17—H17A···O2^iii^	0.93	2.59	3.414 (8)	148

Symmetry codes: (i) −*x*, *y*−1/2, −*z*+1/2; (ii) *x*, *y*+1, *z*; (iii) −*x*, *y*+1/2, −*z*+1/2.
